# Progesterone initiates tendril formation in the oviducal gland during egg encapsulation in cloudy catshark (*Scyliorhinus torazame*)

**DOI:** 10.1186/s40851-023-00211-y

**Published:** 2023-05-30

**Authors:** Koya Shimoyama, Mai Kawano, Nobuhiro Ogawa, Kotaro Tokunaga, Wataru Takagi, Makito Kobayashi, Susumu Hyodo

**Affiliations:** 1 Laboratory of Physiology, Atmosphere and Ocean Research Institute, The University of Tokyo, 5-1-5 Kashiwanoha, Kashiwa, Chiba 277-8564 Japan; 2 Ibaraki Prefectural Oarai Aquarium, Oarai, Ibaraki 311-1301 Japan; 3 Department of Natural Sciences, International Christian University, Tokyo, 181-8585 Japan

**Keywords:** Catshark, Elasmobranchs, Sex steroid, Progesterone, Egg-capsule formation, Collagen secretion, Egg-laying cycle

## Abstract

The diverse reproductive strategies of elasmobranchs (sharks, rays, and skates) have attracted research attention, but the endocrine control of reproduction is still incompletely known in elasmobranchs. By long-term monitoring of the egg-laying cycle in cloudy catsharks (*Scyliorhinus torazame*), we recently demonstrated a transient increase in plasma progesterone (P4) levels just prior to the appearance of the capsulated eggs in the oviducts. In the present study, we examined the in vivo effects of P4 administration in mature female cloudy catsharks. Although no capsulated eggs were observed following the implantation of P4-containing silicone tubing, we did find dark swollen oviducts in the abdominal cavity, in which clumps of long and coiled tendrils were observed. The tendril is an extension of the egg capsule, and the formation of the egg capsule begins with the tendril before main capsule formation. During the period of P4 implantation, the tendrils elongated, and their diameters were significantly increased on day 2 of treatment. Tendril formation was also confirmed on the day of endogenous P4 surge. Tendrils were not formed in catsharks implanted with estradiol-17β or testosterone. Histological analysis of the oviducal gland revealed that P4 implantation induced the secretion of tendril materials from the secretory tubules in the baffle zone, while the tendril materials were stored in the cytoplasm of the secretory cells under low P4 condition. Morphometrically, the area of secreted luminal materials in the secretory tubules was highly correlated to the diameter of tendrils. Our results suggest that the P4 surge during the egg-laying cycle serves as a trigger for egg capsule formation in the oviducal gland of cloudy catshark, but the hormonal signals were incomplete as the main capsule was not formed. Further studies are required to identify the hormones required for ovulation and formation of the main egg capsule.

**Supplementary Information:**

The online version contains supplementary material available at 10.1186/s40851-023-00211-y.

## Background

Reproductive strategies of cartilaginous fishes (sharks, rays, skates, and chimaeras) are unique among piscine vertebrates. All cartilaginous fishes are copulating animals, while external fertilization is predominant in bony fishes. Approximately 60% of elasmobranchs (sharks, rays, and skates) are viviparous, and their reproductive cycles and gestation periods are lengthy, on a time scale of several months or years [1, 2]. Furthermore, after mating, females of many cartilaginous fishes have been reported to store sperm in their reproductive tract for fertilizing the ovulated eggs [3]. In the case of oviparous cloudy catshark, *Scyliorhinus torazame*, females lay two capsulated eggs once every 2 to 4 weeks throughout the year. The ovulated eggs are fertilized using the stored sperm and encapsulated in the oviducal glands. The capsulated eggs are then retained in the lower oviducts for a certain period, such as 1–2 weeks, prior to oviposition. Following oviposition, the female catshark fertilizes the next ovulated eggs using the stored sperm again without mating [4]. These features complicate the research methods for determining the timing of fertilization and subsequent reproductive events, and thus limit progress in the understanding of reproductive physiology in cartilaginous fishes, despite its importance phylogenetically, ecologically, and physiologically.

Sex steroids are critical factors regulating the reproduction of vertebrates. In mammals, estradiol-17β (E2) secretion is stimulated by pituitary follicle-stimulating hormone (FSH) during follicular growth [5]. Plasma E2 levels are relatively low when follicles are small in the ovary [6, 7]. Following the full development of follicles, the increase in plasma E2 induces positive feedback to promote a luteinizing hormone (LH) surge, which triggers oocyte maturation and ovulation [8–10]. After ovulation in mammals, plasma E2 levels decrease, while the corpus luteum secretes progesterone (P4) to maintain pregnancy and suppress gonadotropin releasing hormone (GnRH)/LH pulsatile release [11]. In teleosts, on the other hand, E2 acts on the liver to promote synthesis of vitellogenin, a precursor of yolk protein, and follicular growth [12]. When yolk accumulation is completed, an LH surge occurs to induce secretion of maturation-inducing steroids such as 17α,20β-dihydroxy-4-prognen-3-one (DHP) or 17,20β,21-trihydroxy-4-prognen-3-one (20β-S), the teleost major progestins [13–15]. The maturation-inducing steroids then trigger oocyte maturation [16].

Contribution of sex steroids to reproduction has also been indicated in elasmobranchs [17–21]. Elevation of plasma E2 levels during follicular development was reported in various oviparous and viviparous elasmobranchs [19, 22]. E2 treatment stimulated vitellogenin synthesis [23, 24] and oviduct maturation [25]. In viviparous elasmobranchs, plasma P4 levels were high during early- to mid-pregnancy in spiny dogfish *Squalus acanthias* [26] and bonnethead shark *Sphyrna tiburo* [22], suggesting that P4 contributes to maintenance of pregnancy. In oviparous elasmobranchs, increases in plasma P4 levels were found during the ovulation/egg-encapsulation period [27]. However, endocrine control of ovulation, fertilization, and egg encapsulation mostly remain to be clarified in elasmobranchs, largely due to the above-mentioned difficulties of tracing their reproductive events.

We recently introduced a portable ultrasound device to monitor encapsulated eggs in oviducts and succeeded in assessing the egg-laying cycles of oviparous cloudy catshark *Scyliorhinus torazame* for 6 months [4]. Furthermore, concomitant measurement of plasma sex steroids revealed cyclic patterns of E2, testosterone (T), and P4 levels during the egg-laying cycle [4]. In particular, a surge in plasma P4 was consistently observed 2 days prior to the appearance of capsulated eggs in oviducts. A transient increase in circulating P4 was also reported in little skate, *Leucoraja erinacea* [27], suggesting that the P4 surge may effect ovulation, internal fertilization, and egg-capsule formation in oviparous elasmobranchs.

In the present study, we developed an administration protocol of sex steroids using porous silicone tubing and examined the in vivo effects of P4 surge in female cloudy catshark. We found that the oviducal gland (also known as the egg-shell gland or nidamental gland) produces tendrils without main egg case following the administration of P4. The tendril is a curly whisker-like structure extending from the four corners of the egg-capsule wall, and it helps anchor the egg-capsule to sea grass in natural conditions. Tendril formation is the initial step of egg-capsule formation in lesser spotted dogfish, *Scyliorhinus canicula* [28]. Our findings provide the first direct physiological role of circulating P4 surge in the elasmobranch reproductive cycle.

## Methods

### Animals

Sexually mature female cloudy catsharks (*Scyliorhinus torazame*) were transported from the Ibaraki Prefectural Oarai Aquarium to the Atmosphere and Ocean Research Institute, The University of Tokyo. They were kept in 2000 and 3000 L holding tanks filled with recirculating natural seawater (35‰) at 16ºC under a constant photoperiod (12L:12D) and were fed with chopped anchovy and sardine to satiation twice a week. After acclimating in the holding tanks for at least 2 weeks, experimental fish (total length, 43.1 ± 1.8 cm; body weight, 364.4 ± 41.8 g; mean ± S.E.M.) were transferred to a 1000 L tank with a low water level for ultrasound investigation [4]. Individuals were identified by a tag tied to the dorsal fin. All animal experiments were approved by the Animal Ethics Committee of the Atmosphere and Ocean Research Institute of the University of Tokyo (P19-2). The present study was carried out in compliance with the ARRIVE guidelines.

### Establishment of steroid administration protocol

Sex steroids (E2 [Sigma-Aldrich, St Louis, MO, USA], P4, and T [Wako Pure Chemical Corporation, Osaka, Japan]) were dissolved in ethanol at 100 μg/μl for T and 50 μg/μl for P4 and E2. The dissolved steroids were diluted in sesame oil (Kanto Kagaku, Tokyo, Japan) (1:9 for T, and 1:4 for P4 and E2) to obtain 10 µg/μl solution. The mixtures were left to stand for 48 h without caps to evaporate ethanol at room temperature. Silicone tubing (Silascon tubing; inner and outer diameters 2.0 mm and 3.0 mm, respectively; Kaneka Medics, Osaka, Japan) was cut into lengths of 2 or 8 cm, and they were filled with 50 µl (2 cm tubing, 0.5 mg steroid) or 200 µl (8 cm tubing, 2 mg steroid) steroid solution, respectively. Both ends of the tubing were sealed with silicone adhesive (Shin-Etsu Silicone one component RTV neutral cure system; Shin-Etsu, Tokyo, Japan). For the control group, an 8-cm silicone tubing containing 200 μl of sesame oil was prepared.

Mature female catsharks which had not carried eggs for more than 2 months were used for establishment of the implantation protocol, because they were shown to have low circulating levels of sex steroids. For implantation of the steroid-containing silicone tubing, catsharks were anesthetized with 0.02% (w/v) ethyl 3-aminobenzoate methanesulfonate (Sigma-Aldrich). Blood samples (0.2 ml) were collected from the caudal vasculature using a heparinized syringe connected to a 22-gauge needle. A small incision was made on the left flank of the fish, and the P4-containing silicone tubing was gently inserted into the abdominal cavity. The incision was then closed with nylon thread (No. 3; Matsuda Ika Kogyo, Tokyo, Japan) and a suture needle (No. 1; Igarashi Ika Kogyo, Tokyo, Japan). Fish were recovered from anesthesia by flushing aerated SW over the gills. After the surgery, implanted individuals were kept in separate tanks (60.0 cm × 26.0 cm × 25.5 cm, 30 L), and blood samples were collected 1, 3, 6, 24, 48, and 72 h after the implantation. Following the blood sampling at 72 h, the incision was re-opened, the silicone tubing was removed from the abdominal cavity, and the incision was closed again with nylon thread. Blood samples were collected on days 1 and 2 after the removal of silicone tubing as described above. All blood samples were centrifuged at 10,000 × *g* for 5 min at 4ºC to obtain plasma. 0.1 ml of plasma was used for steroid extraction as described below.

### Steroid implantation experiments

In our previous long-term monitoring of breeding catsharks, plasma P4 levels were nearly undetectable during most of the egg-laying cycle, but levels sharply increased just prior to the ovulation/egg-capsule formation [4]. The P4 levels then gradually decreased to the initial low levels following the appearance of capsulated eggs in the oviducts. Since the egg-carrying period ranged from 11 to 21 days in our previous investigation (17.4 ± 0.4 days on average; [4]), catsharks at 5 to 13 days after the appearance of capsulated eggs were identified by ultrasound investigation using a portable device (Pocket echo Miruco; Nippon Sigmax Co. Ltd., Tokyo, Japan). These individuals are considered to have low endogenous plasma P4 levels, and thus were suitable for steroid implantation experiments. Therefore, all the implanted animals had capsulated eggs in their lower oviducts during the implantation surgery. Implantation of the steroid-containing silicone tubing was conducted as described above. After the implantation, each individual was kept in a separate tank (30 or 250 L) for 1 to 5 days without feeding.

### Blood and tissue sampling

On days 1, 2, and 5 following the steroid implantation, catsharks were anesthetized with 0.02% (w/v) ethyl 3-aminobenzoate methanesulfonate (Sigma-Aldrich), and blood samples were collected as described above. After decapitation, the reproductive organs (ovary, oviducal glands, and oviducts) were dissected out for examining the effects of steroid implantation (P4, *N* = 15; T, *N* = 3; E2, *N* = 4; sesame oil, *N* = 3). In addition to the steroid implantation groups, intact individuals experiencing an endogenous P4 surge were sampled. To determine the endogenous P4 surge, daily ultrasound investigation and blood sampling were conducted in the morning as described previously [4], and plasma P4 levels were measured using a rapid protocol (see below). When a sharp increase in plasma P4 levels was found, the same individual was sampled in the afternoon of the same day (*N* = 4). Furthermore, intact individuals were sampled on the day or 1 day after the appearance of capsulated eggs (*N* = 5). The oviducal glands and oviducts were cut longitudinally and fixed with 4% paraformaldehyde in 50 mM phosphate buffer (pH 7.4) at 4ºC for 2 days. The lengths of tendril and egg capsule were measured.

### Stereomicroscope and scanning electron microscopy observation

The fixed oviducal glands with or without tendrils were rinsed in 70% ethanol. Micrographs of oviducal gland and oviduct were obtained under a stereomicroscope (M165FC; Leica, Wetzlar, Germany) attached to a digital camera (Wraycam-noa630; Wreymer, Osaka, Japan). Diameter of tendril was determined using a digital microscope VHX-7000 and a software package (Keyence, Osaka, Japan).

For scanning electron microscopy observation, the tendril-forming area of the oviducal gland was trimmed to an approximately 1-cm cube, dehydrated in graded concentrations of ethanol, immersed in butyl alcohol, and dried in a vacuum freeze-dryer (JFD-300; JEOL Ltd, Tokyo, Japan) overnight. Tissues mounted on a sample holder were coated with platinum palladium using an ion sputtering apparatus (E-1030; Hitachi High-Technologies Corp., Tokyo, Japan) and were observed using a scanning electron microscope (s-4800; Hitachi High-Technologies Corp.).

### Histological observation

The tendril-forming areas of the fixed oviducal glands from P4- and sesame oil-treated fish were trimmed to an approximately 1-cm cube. Tissues were washed with 70% ethanol, dehydrated in graded concentrations of ethanol, cleared with methyl benzoate, and embedded in Paraplast Plus (Leica Biosystems, Nußloch, Germany). Sections were cut at 8 µm thickness and mounted onto MAS-GP-coated glass slides (Matsunami Glass, Osaka, Japan). Some sections were stained with alcian blue and periodic acid Schiff (AB/PAS) or hematoxylin and eosin (HE). For AB/PAS staining, sections were stained with AB (pH 2.5; Nacalai Tesque, Kyoto, Japan) for 30 s, washed with distilled water (DW), and then oxidized in 0.5% periodic acid solution for 10 min, followed by immersion in Schiff’s reagent (Wako Pure Chemical Corporation) for 15 min. Sections were rinsed in sulfurous acid three times, and then counterstained with Mayer’s hematoxylin (Wako Pure Chemical Corporation). For HE staining, rehydrated sections were treated with Mayer’s hematoxylin for 10 min, washed in running tap water for 15 min, and then stained with eosin (Chroma Gesellschaft Schmid, Koengen, Germany) for 5 min. Stained sections were dehydrated with graded ethanol, cleared in xylene, and mounted with Permount (Thermo Fisher Scientific, Waltham, MA, USA). Micrographs were obtained using a virtual slide system (BZ-X800 and accompanying software; Keyence). The areas of secretory tubule and its lumen were determined using ImageJ software [29].

### Steroid extraction and measurement of P4, T, and E2

Steroid extraction from plasma and measurement of P4, T, and E2 levels were performed following the methods described previously [4, 30]. Briefly, 400 µl of diethyl ether was added to 100 µl plasma in a glass test tube, vortexed, and allowed to separate. The upper diethyl ether layer was recovered, and this extraction procedure was repeated three more times. The diethyl ether from repeated extractions was pooled and dried under a nitrogen stream at 37ºC. Samples were reconstituted with 100 µl of EIA buffer (Cayman Chemical Company, Ann Arbor, MI, USA) and stored at –30 ºC until measurement.

Plasma concentrations of E2, T, and P4 were measured using commercially available ELISA kits (Cayman Chemical Company) following the manufacturer’s instructions [4]. In an ELISA plate pre-coated with secondary antibody, 50 µl of extracted plasma sample was mixed with 50 µl of AChE tracer and 50 µl of primary antibody. The assay plate was then sealed and incubated at 4ºC overnight. After washing five times with wash buffer, 200 µl of Ellman’s reagent was added to each well for color development at 25ºC for 60 to 100 min. For the rapid protocol to monitor the daily P4 levels, the incubation with primary antibody was conducted at 25ºC for 60 min. Absorbance of each well was measured at 405 nm using a microplate reader (Multiskan FC; Thermo Fisher Scientific).

### Statistical analysis

Values are presented as means ± S.E.M. Effects of steroid or vehicle implantation on plasma steroid levels were analyzed by comparing before and after the implantation by paired *t*-test or Wilcoxon test following the normality test, and by comparing with vehicle control by Kruskal–Wallis test followed by Dunn’s multiple comparison test. Plasma steroid levels at the first day of P4 surge were compared with those of the previous day by paired *t*-test. Changes in tendril length and diameter, and changes in areas of tendril components and secretory tubules were analyzed by one-way ANOVA followed by Tukey’s multiple comparison test. Correlation between diameters of tendrils and areas of tendril components, and between diameters of tendrils and areas of secretory tubules were analyzed by Pearson correlation test. Normality of samples was confirmed prior to ANOVA. *P* values < 0.05 were considered statistically significant. All analyses were performed using Prism Ver. 9 for Windows (GraphPad Software, San Diego, CA, USA).

## Results

### Establishment of in vivo steroid administration protocol

**Fig. 1:**
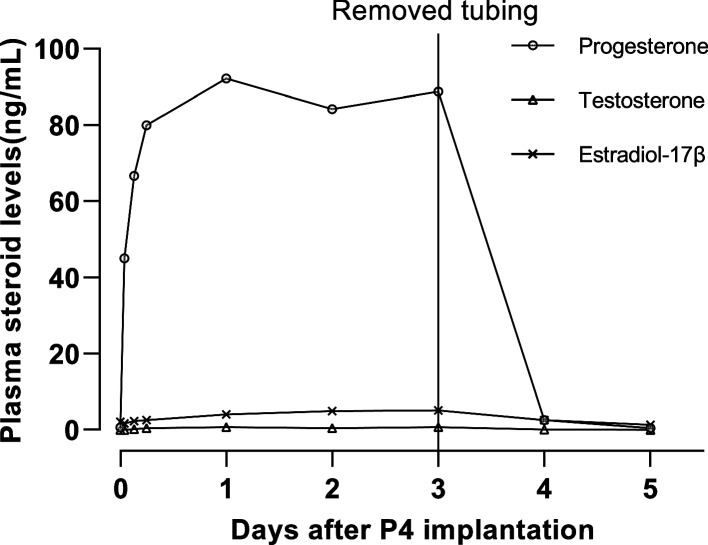
Changes in plasma steroid levels following the implantation of silicone tubing filled with P4. Non-breeding mature female catshark was used in this experiment. Note that plasma P4 level sharply increased following the implantation, while the level rapidly decreased to the initial low level after the removal of silicone tubing

Our previous study showed that the increase in plasma P4 occurs prior to the appearance of capsulated eggs, and the high P4 levels were maintained for 2 days [4]. To reproduce the continuous high circulating P4 levels, we adopted an implantation protocol that was previously used in goldfish, where steroid dissolved in sesame oil was filled in a porous silicone tubing [31]. Circulating level of P4 was less than 1 ng/mL in a non-breeding catshark, and implantation of 8 cm silicone tubing containing P4 increased the circulating level of P4 sharply after 1 h and the level peaked on day 1 (Fig. 1). The high P4 level was maintained for at least 3 days while the implanted tubing remained in the abdominal cavity, but the plasma P4 level rapidly decreased to the initial low level 24 h after the removal of tubing (Fig. 1). The implantation of P4 did not affect plasma levels of T or E2 (Fig. 1).

### Effects of sex steroid implantation

**Fig. 2:**
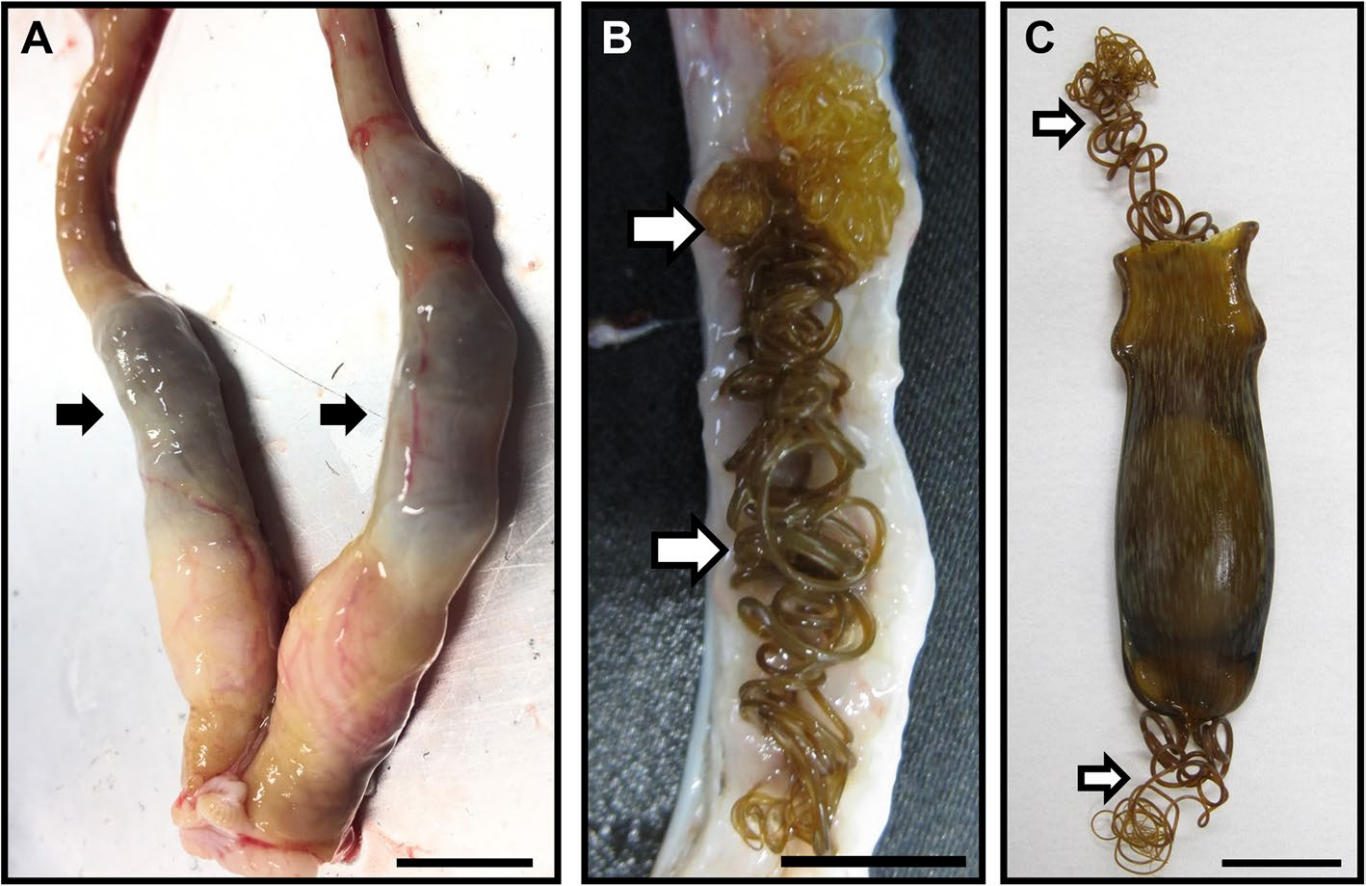
Effects of P4 implantation in the initial experiment. **A** Dark-colored, swollen oviducts were found in the abdominal cavity. **B** Long and coiled tendrils were found in the oviduct. **C** Egg-capsule of cloudy catshark. Arrows indicate tendrils extending from the four corners of egg-capsule. Scale bars, 1 cm

In the initial investigation, we examined effects of P4 administration by monitoring ovulation and encapsulation with non-lethal ultrasound. In this experiment, a breeding catshark was implanted with a P4-containing 8-cm silicone tubing for 2 days followed by removal of the silicone tubing for 5 days to mimic the P4 surge that occurs in a natural egg-laying cycle. However, no obvious change such as appearance of newly encapsulated eggs was observed for 7 days. Therefore, we dissected the implanted individual on day 7, and we found dark, swollen oviducts in the abdominal cavity (Fig. 2A). Dissection of oviducts revealed that the dark-colored materials inside the oviducts were tendrils (Fig. 2B, C). Besides the long and coiled tendril, no egg or egg-capsule wall were found in the oviducts (Fig. 2B).

In the second experiment, catsharks at low endogenous plasma P4 were implanted with P4 silicon tubing and then sampled 1, 2, and 5 days thereafter. Plasma P4 levels were significantly increased by the P4 implantation (Table 1). No changes were observed in plasma E2 and T levels after the P4 treatment, although plasma E2 levels tended to decrease following the implantation of P4 for 5 days (Table 1). As the vehicle control, a sesame oil-containing tubing was implanted into the abdominal cavity. No changes were observed in plasma P4, E2, or T levels by vehicle treatment for 2 days. Plasma P4 levels of P4-implanted individuals (days 1 and 2) were significantly higher than those of vehicle-treated individuals (Table 1). The increased P4 levels ranged from 42 ng/mL to 98.8 ng/mL (Supplementary Table 1), except for individual No. 7 (163.9 ng/mL). The elevated levels were within the physiological range as the plasma P4 levels can be increased to at least 75 ng/mL under the natural P4 surge [4].

**Table 1:** Plasma steroid levels before and after steroid (P4, T, E2) or vehicle (V) implantation

	P4 (ng/mL)		T (ng/mL)		E2 (ng/mL)		Tendril
	Before	After	Before	After	Before	After	
P4 1 day	0.6 ± 0.3	82.2 ± 3.7 ^***, †^	1.8 ± 0.6	3.4 ± 0.9	18.7 ± 7.3	16.5 ± 5.8	4/4
P4 2 days	1.6 ± 0.6	92.5 ± 17.0 ^**, †^	5.6 ± 2.2	5.0 ± 1.9	29.7 ± 7.9	22.0 ± 5.9	5/5
P4 5 days	3.0 ± 1.6	53.7 ± 6.0 ^*^	12.0 ± 7.7	10.6 ± 6.4	30.5 ± 9.1	10.1 ± 4.5	3/3
P4 2 days 2-cm tubing	0.5 ± 0.2	23.4 ± 6.9	7.1 ± 2.6	3.7 ± 0.9	25.2 ± 6.0	15.5 ± 4.5	3/3
T 2 days	1.1 ± 0.0	0.9 ± 0.3	10.9 ± 5.2	161.1 ± 39.0	24.4 ± 12.5	29.0 ± 11.0	0/3
E2 2 days	4.4 ± 0.1	3.1 ± 2.4	8.4 ± 1.8	10.1 ± 1.8	31.1 ± 3.2	54.2 ± 3.5 ^**^	1/4
V 2 days	1.2 ± 0.1	1.1 ± 0.4	7.3 ± 3.3	8.0 ± 3.2	18.5 ± 2.3	15.5 ± 2.2	0/3

Values are expressed as mean ± S.E.M. *, **, and *** denote significant differences in plasma steroid levels before and after the implantation (*P* < 0.05, 0.01, and 0.001, respectively). † denotes significant difference from vehicle control (*P* < 0.05)

**Fig. 3:**
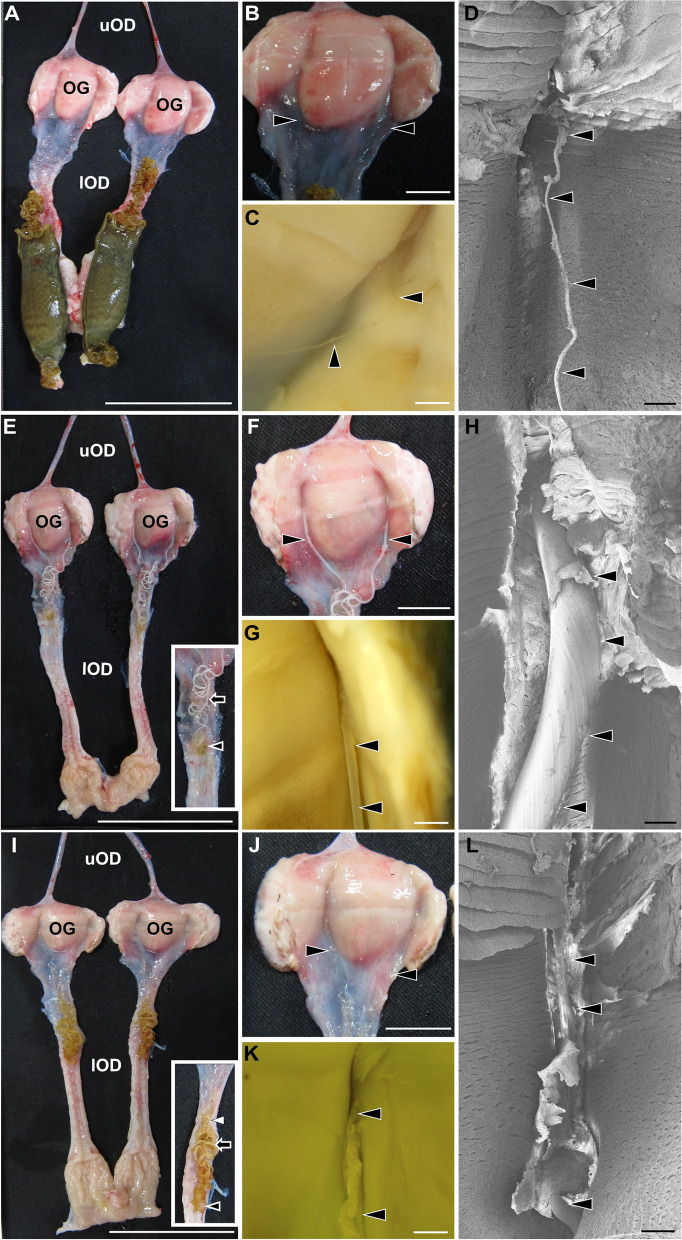
Tendril formation in the oviducal gland after P4 implantation. Day 1 (**A–D**), day 2 (**E–H**), and day 5 (**I–L**) of P4 implantation. **A, E, I** The whole view from the oviducal gland (OG) to the upper (uOD) and lower (lOD) oviducts. The oviducal gland and the lower oviduct were cut longitudinally to expose formed tendrils. Black arrowheads, black arrows, and white arrowhead represent the initial thin portion, the middle thick portion, and the posterior thin portion of the formed tendrils (insets in **E** and **I**) (**B, F, J**) Magnified view of the oviducal gland. Tendrils (arrowheads) were extended from the middle portion of oviducal glands. **C, G, K** Stereomicroscope images of the formed tendrils (arrowheads). **D, H, L** Observation of the formed tendrils (arrowheads) using a scanning electron microscope. Note that the diameter of tendrils considerably increased on day 2 (**H**), and then decreased on day 5 (**L**). Scale bars, 5 cm (**A, E, I**), 1 cm (**B, F, J**), 1 mm (**C, G, K**) and 300 µm (**D, H, L**)

**Fig. 4:**
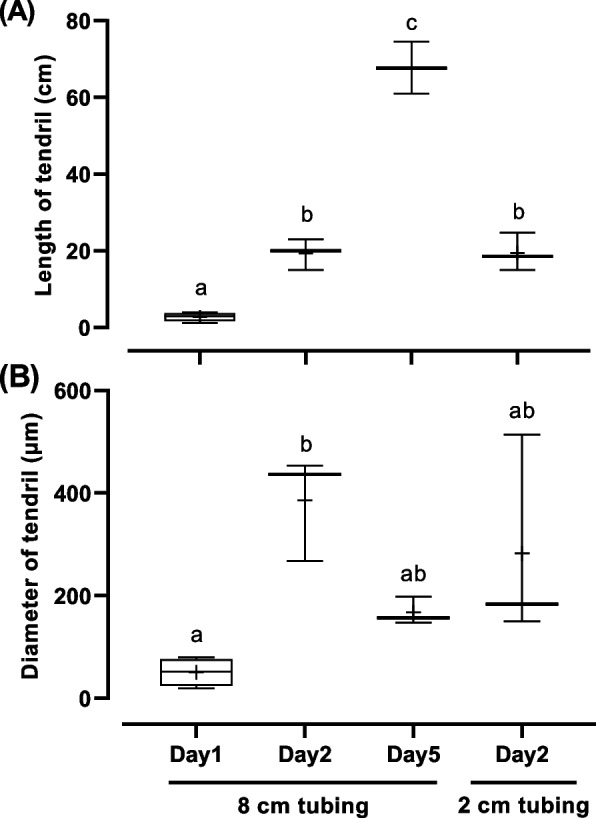
Changes in length (**A**) and diameter (**B**) of tendrils following the implantation of P4. Different letters denote significant differences in length and diameter (*P* < 0.05)

In all P4-treated catsharks, tendrils were commonly observed forming from the middle portion of the oviducal gland (Fig. 3). Meanwhile, the length and diameter of tendrils altered with duration of implantation (Figs. 3 and 4). On the first day of P4 implantation, the formed tendrils were too thin (51.0 ± 11.8 µm) to be observed without a microscope (Fig. 3B–D). On day 2, the tendrils greatly thickened to 385.9 ± 48.4 µm (Figs. 3F–H and  4B), and elongated into the oviducts (Figs. 3E, F and 4A). The tendrils were further elongated on day 5; the long and coiled tendrils were observed in the oviducts (Figs. 3I and 4A). On the other hand, the diameter of tendrils decreased on day 5 and returned to levels similar to those on day 1 (Figs. 3J–L and  4B). Consequently, the tendrils on day 5 were composed of three parts: the initial thin portion (black arrowheads in Fig. 3E and I insets), the middle thick portion (black arrows), and the posterior thin portion (white arrowhead). Tendril formation was not observed in vehicle-treated individuals.

To examine dose-dependent effects of P4 implantation, a shorter 2-cm silicone tubing was used. After 2 days of implantation, plasma P4 levels increased to 23.4 ± 6.9 ng/mL, which corresponds to approximately one-fourth the P4 concentration in individuals implanted with 8-cm tubing for 2 days (Table 1). Tendril formation was also observed in those individuals implanted with 2-cm P4 tubing (Table 1). The length of tendrils was similar to that of individuals implanted with 8-cm tubing for 2 days (Fig. 4A). The tendril diameter of individuals implanted with 2-cm tubing tended to be smaller than that of individuals implanted with 8-cm tubing, although there was no significant difference due to the large variation (Fig. 4B).

Effects of T and E2 were examined by implantation of 8-cm silicone tubing containing T or E2 for 2 days (Table 1). Plasma T levels before the implantation were 10.9 ± 5.2 ng/mL. Plasma T levels increased 15-times after the implantation, while no change was observed in plasma P4 or E2 levels (Table 1). Tendril formation was not observed in any T-treated catsharks (Table 1).

Plasma E2 levels were significantly increased by the E2 implantation (Table 1). No changes in plasma P4 or T levels were observed following the E2 treatment. Among four individuals examined, only one individual (No. 25) was found to have tendrils in the oviducal glands and oviducts (Table 1, Supplementary Table 1). The plasma P4 level of individual No. 25 was 15.4 ng/mL even prior to the E2 implantation (11.3 ng/mL after E2 implantation; Supplementary Table 1). This concentration was higher than that in individual No. 13, in which 2-cm P4-containing silicon tubing was implanted for 2 days (8.9 ng/mL; Supplementary Table 1), and thus the tendril formation may not have been related to implantation in this case.

**Fig. 5:**
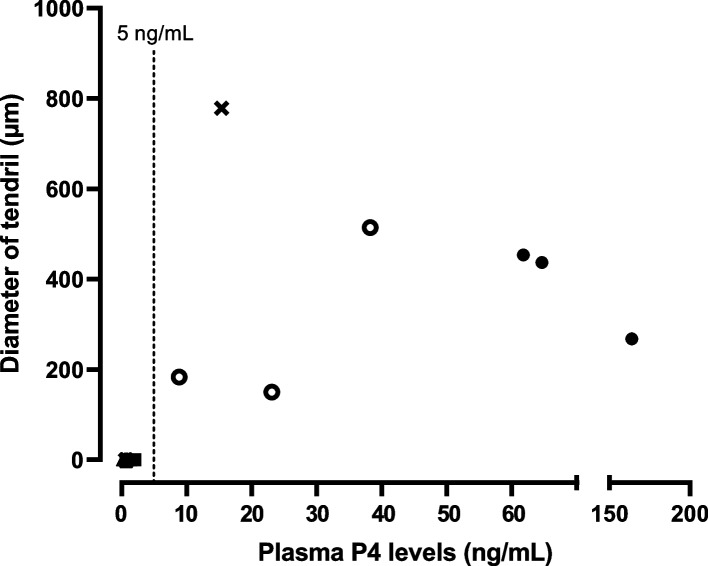
Distribution graph showing diameter of tendrils and plasma P4 levels of steroid- or vehicle-treated individuals. Filled circles, P4 implantation for 2 days using 8 cm tubing (*N* = 3); open circles, P4 implantation for 2 days using 2 cm tubing (*N* = 3); cross marks, E2 implantation for 2 days (*N* = 4); filled triangles, T implantation for 2 days (*N* = 3); filled squares, vehicle implantation for 2 days (*N* = 3). Because individuals implanted with E2, T, and vehicle (with the exception of one E2-implanted individual) had plasma P4 levels less than 3 ng/mL and did not form tendrils, these marks overlap and cannot be individually distinguished in this figure

No significant correlation was detected between plasma P4 levels and tendril diameter (Fig. 5; *r* = 0.19, *P* = 0.088). All catsharks with plasma P4 levels over 8 ng/mL produced tendrils, while vehicle-, T-, or E2-implanted individuals, in which plasma P4 levels were less than 3 ng/mL, did not produce tendrils.

### Tendril formation during naturally induced P4 surge

**Fig. 6:**
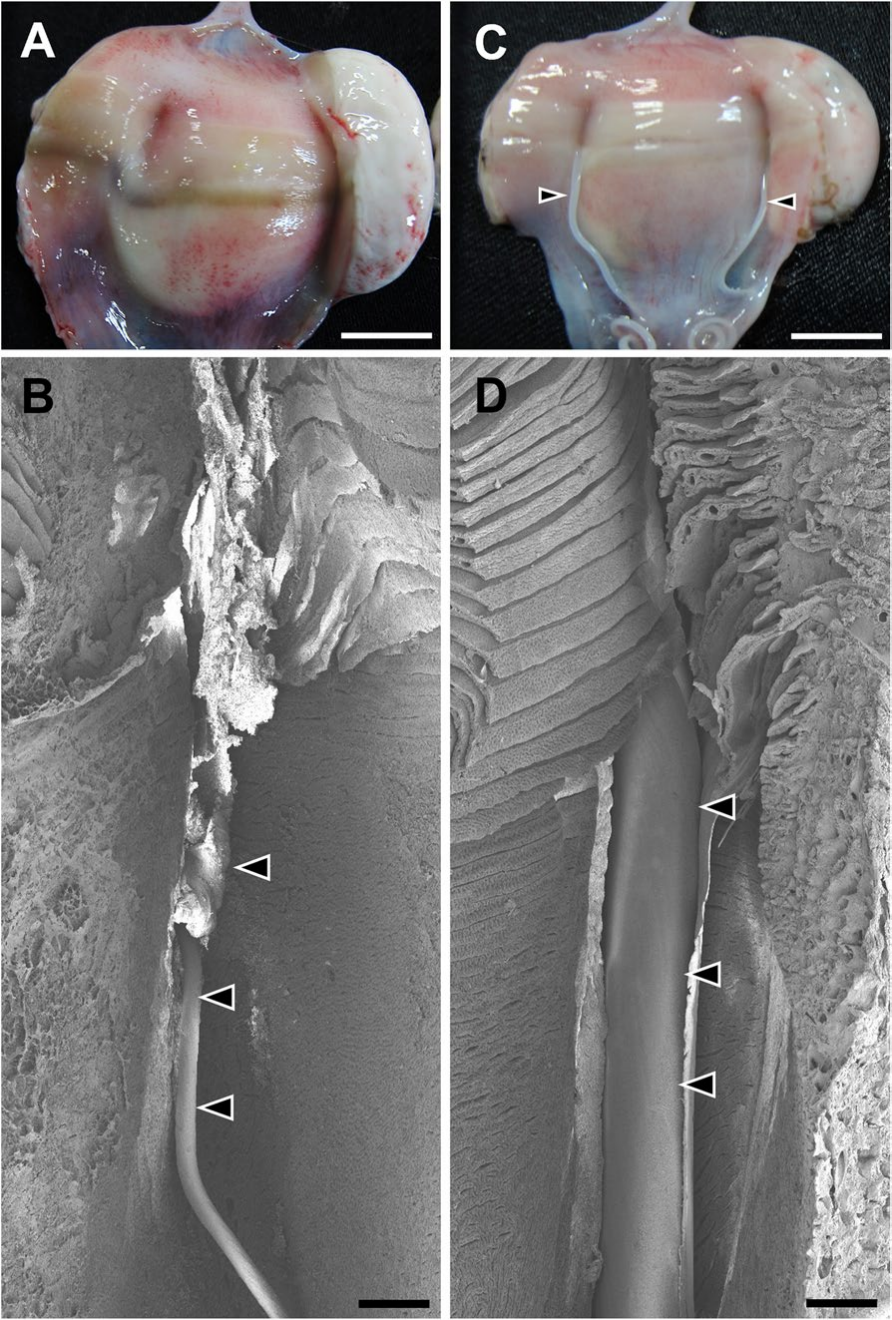
Tendril formation in the oviducal gland during the naturally-progressing egg-laying cycle. **A, B** The individual representing the first day of P4 surge. **C, D** The catshark in which capsulated eggs appeared in the oviducts. This individual probably represents the third day of P4 surge. **A, C** Magnified view of the oviducal gland. Tendrils (arrowheads) were extended from the middle portion of oviducal glands. **B, D** Observation of the formed tendrils (arrowheads) using a scanning electron microscope. Scale bars, 1 cm (**A, C**) and 300 µm (**B, D**)

As we found that P4 treatment induces tendril formation, we examined the tendril formation in the oviducal glands among the catsharks under the endogenous P4 surge during the natural egg-laying cycle. To determine the beginning of the P4 surge, we conducted daily P4 measurement. The plasma P4 concentration on the first day of P4 surge was 27.1 ± 2.0 ng/mL (*N* = 4), which was significantly higher (*P* < 0.01) than that on the previous day (0.7 ± 0.2 ng/mL) (Supplementary Table 2). Among these individuals, thin (43.2 ± 7.1 µm), short tendrils were found in the oviducal glands (Fig. 6A, B). These tendrils had characteristics similar to those of catsharks implanted with P4 for 1 day (diameter, 51.0 ± 11.8 µm; Fig. 4B). Except for the tendrils, egg capsules or ovulated eggs were not found in the reproductive tracts on the first day of endogenous P4 surge.

In addition to sampling on the first day of endogenous P4 surge, sampling of catsharks was conducted on the day the capsulated eggs were found in the oviducts or on the day after. Our previous study indicated that capsulated eggs appear in the oviducts approximately 3 days after the onset of P4 surge [4], and therefore we sampled the individuals 3 or 4 days after the onset of endogenous P4 surge. In these individuals, thick tendrils were found to be extended from the oviducal glands (Fig. 6C, D), and connected to the egg capsule with fertilized eggs in the oviducts. The thickness of tendrils (453.5 ± 150.8 µm; Supplementary Table 2) was similar to that in individuals implanted with P4 for 2 days (385.9 ± 48.4 µm; Fig. 4B).

### Histological observation of tendril-forming area in oviducal glands

**Fig. 7:**
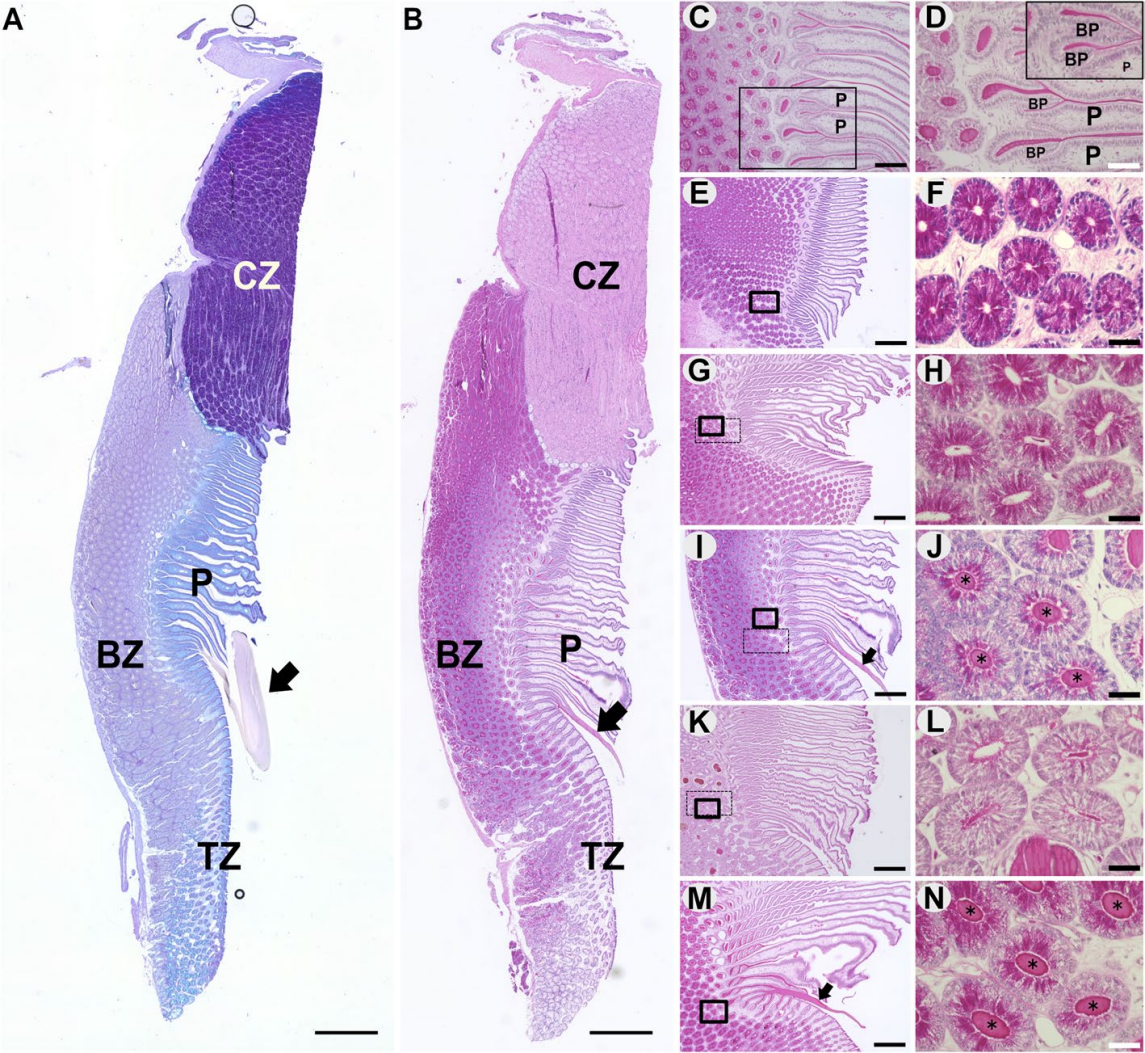
Histological investigation of oviducal glands. **A, B** Sagittal sections stained with AB/PAS (**A**) and HE (**B**). CZ, club zone; BZ, baffle zone; TZ, terminal zone; P, plateaus. **C, D** Magnified view of baffle plates (BP) and plateaus (P) of the section in (**B**). **E** to **N** Posterior portion of the baffle zone (**E, G, I, K, M**) and magnified views of secretory tubules (**F, H, J, L, N**). **E, F** Vehicle treatment for 2 days. (**G, H**) P4 administration for 1 day using 8 cm tubing. (**I, J**) P4 administration for 2 days using 8 cm tubing. (**K, L**) P4 administration for 5 days using 8 cm tubing. **M, N** The individual having the capsulated eggs in the oviducts, which probably represents the third day of P4 surge. Square frames with solid line represent the magnified areas in (**F, H, J, L, N**). The areas of tendril components (*) and secretory tubules in the square frames with dotted line were measured in Fig. 8. Arrows indicate tendrils. Scale bars,1 mm (**A, B**), 200 µm (**C**), 100 µm (**D**), 500 µm (**E, G, I, K, M**), and 50 µm (**F, H, J, L, N**)

The oviducal gland of cloudy catshark could be divided into at least three zones based on histochemical characteristics: 1) club zone, 2) baffle zone, and 3) terminal zone (Fig. 7A, B). The club zone was intensely stained with PAS. The club zone and the terminal zone were located in the rostral and caudal parts of the oviducal gland, respectively (Fig. 7A, B). Meanwhile, the baffle zone was situated in the middle portion of the oviducal gland with numerous baffle plates and plateaus (Fig. 7A, B). The baffle zone was densely filled with secretory tubules, which were intensely stained with eosin (Fig. 7B, E, F). In the baffle zone of P4-implanted individuals, eosin-positive components were secreted from the interspaces between the baffle plates (Fig. 7C, D) at the posterior-most part of the baffle zone and passed through the spaces between the plateaus (Fig. 7C, D) to form an eosinophilic tendril (arrows in Fig. 7B, I).

A striking feature of P4-implanted individuals was the large amount of eosinophilic materials that were secreted into the lumen of secretory tubules in the baffle zone (Fig. 7J). On day 1 of P4 implantation, small amounts of eosinophilic materials were found in the lumen of some tubules (Fig. 7H). At this stage, secretory cells were intensely stained with eosin, but the stainability of cytoplasm by eosin decreased at the basolateral side (Fig. 7H). Such a phenomenon was not observed in vehicle-implanted individuals (Fig. 7F).

On the second day of P4 implantation, the lumina of secretory tubules were filled with large amounts of eosinophilic materials (asterisks in Fig. 7J). The lumina are connected to the spaces between baffle plates (Figs. 7C, D), indicating that the eosinophilic material is composing the tendril. At this stage, the intensity of eosin staining in the secretory cells was remarkably decreased; intense eosin staining was evident only in the apical side of the secretory cells (Fig. 7J). On day 5, the amount of eosinophilic materials was decreased both in the lumen and in secretory cells (Fig. 7L).

Large amounts of eosinophilic materials were also found in the lumina of secretory tubules at the baffle zone of intact individuals on the first day of egg-carrying (Fig. 7M, N), which likely corresponded to the stage 3 days after the P4 surge. In the secretory cells of these individuals, intense eosin staining was found only at the apical region of cytoplasm (Fig. 7N).

**Fig. 8:**
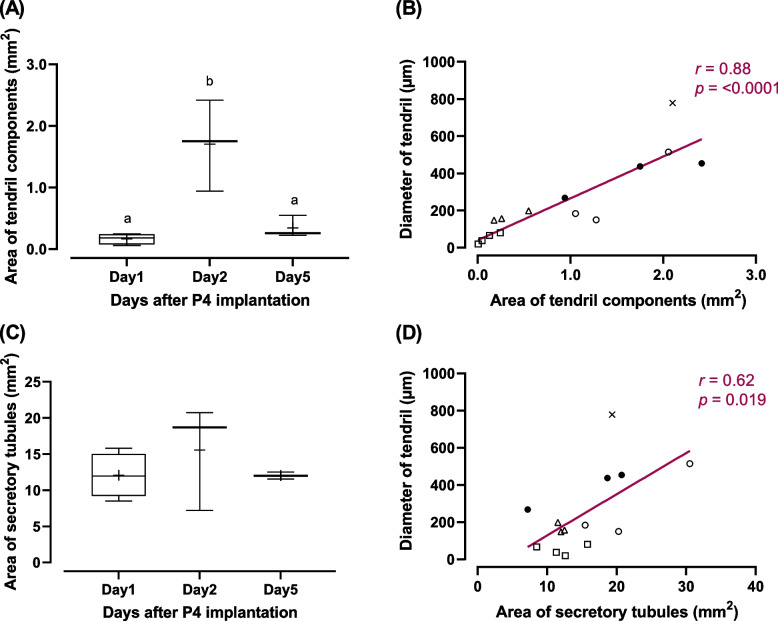
Morphometrical analyses of secretory tubules of baffle zone and tendril components in the secretory lumen. Changes in areas of tendril components (**A**) and secretory tubules (**C**) following the P4 implantation. Strong positive correlation was found between the tendril diameter and the area of tendril components (**B**), while only moderate correlation was observed between the tendril diameter and the area of secretory tubules (**D**); open squares, P4 implantation for 1 day; filled circles, P4 implantation for 2 days using 8 cm tubing; open triangles, P4 implantation for 5 days; open circles, P4 implantation for 2 days using 2 cm tubing; cross marks, E2 implantation for 2 days (one individual formed tendrils). Different letters denote significant differences (*P* < 0.05)

As the histochemical results showed that the secretion of tendril materials (eosinophilic materials) changed depending on the length of P4 implantation, the area of eosinophilic materials in the lumen was measured. The area of eosinophilic materials significantly increased on day 2 and then decreased on day 5 after the P4 implantation (Fig. 8A). The area of eosinophilic materials was highly correlated to the diameter of tendrils (Fig. 8B). Meanwhile, the area of secretory tubules was not significantly different among all of the time points (Fig. 8C). A moderate level of correlation was detected between the area of secretory tubules and the diameter of tendrils (Fig. 8D).

## Discussion

**Fig. 9:**
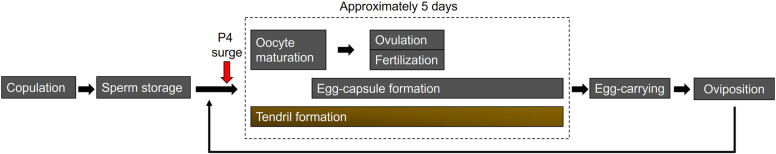
Schematic diagram representing the egg-laying cycle of cloudy catshark. Note that oocyte maturation, ovulation, fertilization, and tendril/egg-capsule formation occur during approximately 5 days following the P4 surge. The present study revealed that P4 administration induces tendril formation. Following egg-laying, females can fertilize the next ovulated eggs using stored sperm without further mating

In the present study, we demonstrated for the first time that P4 implantation induces tendril formation in vivo in cloudy catshark. Recently, we revealed that a P4 surge occurs prior to egg capsule formation in the egg-laying cycles of catshark by concomitant monitoring of the egg-laying cycle with ultrasound and measuring of plasma sex steroids [4] (Fig. 9). Increases in plasma P4 levels prior to ovulation/egg capsule formation were also suggested for several other elasmobranchs [27, 32, 33], and thus we considered that P4 likely contributes to oocyte maturation, ovulation, and egg capsule formation [4]. We further demonstrated that tendril formation occurs on the first day during the naturally induced endogenous P4 surge, prior to ovulation and fertilization. In lesser spotted dogfish, the process of egg capsule formation was investigated in detail; the initial step of egg capsule formation is the formation of posterior tendrils [34, 35]. Taken together, these facts indicate that the P4 surge during the egg-laying cycle in catshark most likely initiates the egg-capsule formation in the oviducal gland (Fig. 9).

Tendril formation was not induced by T or E2 implantations except in one E2-implanted individual. Plasma steroid measurements showed that the plasma P4 level of this individual (No. 25) was 15.4 ng/mL prior to the E2 implantation, and this P4 value is higher than average basal value of plasma P4. These results suggest that this individual (No. 25) was at the stage of endogenous P4 surge before E2 implantation, and possessed sufficient P4 to induce tendril formation. In immature lesser spotted dogfish, Dodd and Goddard [25] reported that long-term treatment with E2 (about 6 months) induced large production of tendrils in the oviducts. Although plasma steroid levels were not measured in the study of lesser spotted dogfish, plasma P4 levels might have increased at some points during the prolonged E2 administration, which stimulated the tendril formation. Collectively, the tendril-inducing function is likely specific to P4, and further studies are required to confirm whether the P4-dependent tendril formation is conserved in other elasmobranchs.

In oviparous little skates, the occurrence of unusual egg-capsule morphology (i.e., unusually long anterior horns) was reported in a P4-treated individual [36]. The horns of little skate are ornamental parts of the egg capsule extending from the four corners of the egg capsule wall [37]. Based on the structural similarity, the elongated horns of the P4-treated little skate are similar to the tendrils in P4-implanted cloudy catshark. The similar results between Selachii (sharks) and Batoidea (rays and skates) indicate that P4 could be commonly involved in the egg capsule formation in oviparous elasmobranchs.

### Secretion of tendril-forming materials in the oviducal gland stimulated by P4

The oviducal gland is the organ responsible for egg capsule formation and is composed of multiple regions with different functions [38–40]. In the present study, at least three regions (club zone, baffle zone, and terminal zone) could be clearly distinguished in the oviducal gland of cloudy catshark. The club zone secretes egg jelly to surround the fertilized egg in the egg capsule [41, 42], while the terminal zone was shown to be the location for sperm storage [41, 43]. On the other hand, the baffle zone (or “D zone” by Rusaouen [39]) is filled with numerous secretory tubules. The major component of elasmobranch egg capsules including tendrils is collagen, and the secretory tubules in the baffle zone were reported to secrete collagen to form the egg capsule [35, 44–46]. In the present study, the baffle zone of catshark was intensely stained with eosin. Eosin is an acidic dye and stains collagen [47, 48], and the tendrils were eosinophilic (Fig. 7B).

Following the P4 implantation, eosinophilic materials were secreted into the lumen of secretory tubules in the baffle zone. The sagittal view of secretory tubules showed that the tubules were connected to the baffle plates and plateaus in the caudal part of the baffle zone (Fig. 7B–D). The eosinophilic materials were secreted from the space between the baffle plateaus to the lumen of the oviducal gland to form a tendril. Eosinophilic materials were sparse in the secretory lumen on day 1 after P4 implantation, and significantly increased on day 2, a pattern that was positively correlated with the changes in tendril diameters. Therefore, our results suggest that P4 induces tendril formation by stimulating the secretion of collagen from the epithelial cells of secretory tubules in the baffle zone.

The collagen secretory process was examined by electron microscopy in lesser spotted dogfish [35, 49, 50]. Collagen is stored in vesicles in the epithelial cells of the secretory tubules. The vesicles then migrate to the apical pole and are secreted into the lumen of the secretory tubule by exocytosis. In the present study, eosinophilic materials were distributed evenly in the cytoplasm of epithelial cells of vehicle-implanted individuals. With P4 implantation, eosin-positive staining was decreased at the basolateral region of epithelial cells on day 1. On day 2, eosin-positive areas were further decreased and were limited to the apical region of the epithelial cells, indicating that P4 stimulated the merocrine secretion of stored collagen vesicles into the lumen and intracellular transport of vesicles from basolateral to apical regions. On day 5 of the P4 implantation, weak eosin signals were found only in the vicinity of the apical pole of epithelial cells, suggesting that the secretory cell was in a “spent” status, in which the production rate could not catch up to the releasing rate.

### Why did P4 only induce tendril formation?

In the present study, P4 stimulation only induced tendril formation. This result was unexpected because P4 surge occurs prior to ovulation, fertilization, and egg capsule formation (Fig. 9). Stimulation of follicular rupture by P4 was reported in birds at ovulation [51]. P4 or progestins (DHP and 20β-s) induce oocyte maturation in amphibians and teleosts, respectively [15, 52, 53]. In lizard, a reduction in P4 levels by removal of corpora lutea resulted in reduced egg weight and disrupted eggshell formation [54]. In lesser spotted dogfish, Feng and Knight [35] reported that egg capsule formation commences with the production of the tips of posterior tendrils at the tendril-forming regions in the baffle zone of oviducal gland, and that activation of the tendril-forming regions spreads toward the anterior direction in the baffle zone to form the thicker parts of tendrils. The production of the tips and the thicker parts of tendrils was similar to our results of day 1 and day 2 of P4 treatments, respectively. In the natural egg capsule formation, the wave of activation was considered to spread to regions responsible for capsule wall formation [35]. It is possible that the tendril-forming region and the capsule-wall-forming region respond to P4 differently, and thus we are interested in exploring the expression and localization of P4 receptor(s) within the baffle zone of the oviducal gland in the future. Although both genomic and non-genomic pathways via various types of P4 receptors are known in vertebrates [55, 56], gene repertories and expression patterns of P4 receptors have not been examined in elasmobranchs.

Our results suggested that other factors (hormones) may be required for the formation of the entire egg capsule and ovulation. During the natural egg-laying cycles of cloudy catshark [4] and little skate [27], the occurrence of the P4 surge was preceded by a decrease in plasma T level, while plasma E2 levels did not show significant fluctuation during the egg-laying cycle. In the present study, the P4 treatment tended to lower plasma E2 levels, while plasma T was maintained at high levels during the P4 implantation. Although E2 and T implantations alone had no effects on ovulation or egg capsule formation, appropriate combinations of P4, E2, and T may be crucial for the formation of the entire egg capsule and ovulation. Further investigations including simultaneous administration of steroids in vivo or in vitro are expected to clarify their roles. In addition, the LH surge triggers oocyte maturation and ovulation in vertebrates [16, 57]. In cartilaginous fishes, fluctuations of FSH and LH during reproductive cycles remain to be clarified, including a possible relationship between LH and P4 surge. Synthesis or purification of homologous FSH and LH will be an important step to pave the ways for physiological studies of the hypothalamo-pituitary–gonadal axis in cartilaginous fishes.

During the natural egg-laying cycles, a transient and significant rise in plasma P4 was observed 2 days prior to the appearance of capsulated eggs in the oviducts [4]. Therefore, we postulated that the transient rise in plasma P4 may be important for the formation of the egg capsule. Some may argue that the decrease of plasma P4 was related to the capsule formation. However, in the initial experiment, the P4 implantation for 2 days followed by removal did not produce the entire egg capsule (Figs. 1 and 2), implying that the decrease in plasma P4 is not a stimulus for egg capsule formation. To confirm this idea, detailed investigation using a 2-cm tubing would be preferable in the future, because the use of 8 cm tubing led to slightly higher plasma P4 levels than those of the natural P4 surge (75 ng/mL) (Supplementary Tables 1 and 2; [4]).

In mammals, P4 secreted from the corpus luteum is important for the proliferation and thickening of endometrium and the maintenance of pregnancy [58, 59]. In viviparous elasmobranchs, plasma P4 levels are high in early- to mid-gestation, implying that P4 may also be important in the maintenance of pregnancy in elasmobranchs [22, 26]. On the other hand, P4 treatment accelerated the time to oviposition, and the egg capsule under P4 stimulation was much darker than normal egg capsule, suggesting that P4 may affect capsulated egg retention and tanning [36, 60]. In the present study, six out of the 11 individuals (54.5%) laid eggs within 2 days following the implantation of P4 for 2 or 5 days, while no individuals laid eggs following the implantation of T or vehicle for 2 days (six individuals) (Supplementary Table 3). With regard to E2 implantation for 2 days, two out of four individuals laid eggs on day 2, and one of them had high plasma P4 levels (Supplementary Table 3). Therefore, our results also suggest that P4 might act on the oviducts and accelerate oviposition in cloudy catshark.

## Conclusion and perspectives

We revealed here for the first time that the elevated P4 during the egg-laying cycle induces tendril formation, the initial step of egg capsule formation in cloudy catshark (Fig. 9). Recent overexploitation and global environmental changes have seriously affected the abundance of oceanic sharks and rays, bringing approximately one-third of shark and ray species into threat of extinction [61]. Therefore, knowledge of elasmobranch reproductive physiology is essential to guide future strategies for conservation of elasmobranchs. Although further studies are necessary for unraveling the mechanisms regulating the various steps from oocyte maturation to egg encapsulation, the present results are a significant achievement in understanding the endocrine control and mechanisms of reproductive events in elasmobranchs.

## Supplementary Information

**Additional file 1: Table S1.** Plasma steroid levels of hormone-implanted individuals and morphometry of formed tendrils.

**Additional file 2: Table S2.** Plasma steroid levels of individuals sampled during the endogenous P4 surge and diameters of formed tendrils.

**Additional file 3: Table S3.** Occurrence of egg-laying in each individual during implantation.

## Data Availability

Relevant data are available from the corresponding author upon reasonable request.

## References

[1] Compagno LJV (1990). Alternative life-history styles of cartilaginous fishes in time and space. Environ Biol Fishes.

[2] Dulvy NK, Reynolds JD (1997). Evolutionary transitions among egg-laying, live-bearing and maternal inputs in sharks and rays. Proc R Soc B.

[3] Wourms JP (1977). Reproduction and development in chondrichthyan fishes. Amer Zool.

[4] Inoue T, Shimoyama K, Saito M, Wong MK, Ikeba K, Nozu R (2022). Long-term monitoring of egg-laying cycle using ultrasonography reveals the reproductive dynamics of circulating sex steroids in an oviparous catshark. Scyliorhinus torazame Gen Comp Endocrinol.

[5] Kanda S (2019). Evolution of the regulatory mechanisms for the hypothalamic-pituitary-gonadal axis in vertebrates – hypothesis from a comparative view. Gen Comp Endocrinol.

[6] Brenner RM, West NB (1975). Hormonal regulation of the reproductive tract in female mammals. Annu Rev Physiol.

[7] Drummond AE, Findlay JK (1999). The role of estrogen in folliculogenesis. Mol Cell Endocrinol.

[8] Legan SJ, Allyn Coon G, Karsch FJ (1975). Role of estrogen as initiator of daily LH surges in the ovariectomized rat. Endocrinology.

[9] Moenter SM, Caraty A, Karsch FJ (1990). The estradiol-induced surge of gonadotropin-releasing hormone in the ewe. Endocrinology.

[10] Xia L, Van Vugt D, Alston EJ, Luckhaus J, Ferin M (1992). A surge of gonadotropin-releasing hormone accompanies the estradiol-induced gonadotropin surge in the rhesus monkey. Endocrinology.

[11] Zalanyi S (2001). Progesterone and ovulation. Eur J Obstet Gynecol Reprod.

[12] Lubzens E, Young G, Bobe J, Cerdà J (2010). Oogenesis in teleosts: how fish eggs are formed. Gen Comp Endocrinol.

[13] Kobayashi M, Aida K, Hanyu I (1987). Hormone changes during ovulation and effects of steroid hormones on plasma gonadotropin levels and ovulation in goldfish. Gen Comp Endocrinol.

[14] Kobayashi M, Aida K, Hanyu I (1988). Hormone changes during the ovulatory cycle in goldfish. Gen Comp Endocrinol.

[15] Nagahama Y, Yamashita M (2008). Regulation of oocyte maturation in fish. Dev Growth Differ.

[16] Yaron Z, Levavi-Sivan B, Farrell AP (2011). Endocrine regulation of fish reproduction. Encyclopedia of Fish Physiology: From Genome to Environment.

[17] Callard IP, Klosterman LL, Sorbera LA, Fileti LA, Reese JC (1989). Endocrine regulation of reproduction in elasmobranchs: Archetype for terrestrial vertebrates. J Exp Zool.

[18] Callard IP, Etheridge K, Giannoukos G, Lamb T, Perez L (1991). The role of steroids in reproduction in female elasmobranchs and reptiles. J Steroid Biochem Mol Biol.

[19] Koob TJ, Callard IP (1999). Reproductive endocrinology of female elasmobranchs: Lessons from the little skate (*Raja erinacea*) and spiny dogfish (*Squalus acanthias*). J Exp Zool.

[20] Awruch CA. Reproduction strategies. In: Shadwick RE, Farrell AP, Brauner CJ, editors. Fish Physiology: Physiology of Elasmobranch Fishes: Structure and Interaction with Environment. San Diego: Academic Press; 2016. 34A:255–310.

[21] Becerril-García EE, Arellano-Martínez M, Bernot-Simon D, Hoyos-Padilla EM, Galván-Magaña F, Godard-Codding C (2020). Steroid hormones and chondrichthyan reproduction: physiological functions, scientific research, and implications for conservation. PeerJ.

[22] Manire CA, Rasmussen LEL, Hess DL, Hueter RE (1995). Serum steroid hormones and the reproductive cycle of the female bonnethead shark. Sphyrna tiburo Gen Comp Endocrinol.

[23] Craik JCA (1978). The effects of oestrogen treatment on certain plasma constituents associated with vitellogenesis in the elasmobranch *Scyliorhinus canicula* L. Gen Comp Endocrinol.

[24] Prisco M, Valiante S, Maddalena Di Fiore M, Raucci F, Del Giudice G, Romano M, et al. Effect of 17β-estradiol and progesterone on vitellogenesis in the spotted ray *Torpedo marmorata* Risso 1810 (Elasmobranchii: Torpediniformes): Studies on females and on estrogen-treated males. Gen Comp Endocrinol. 2008;157(2):125–32. 10.1016/j.ygcen.2008.04.011 18555067

[25] Dodd JM, Goddard CK (1961). Some effects of oestradiol benzoate on the reproductive ducts of the female dogfish *Scyliorhinus caniculus*. Proc Zool Soc Lond.

[26] Tsang PCW, Callard IP (1987). Morphological and endocrine correlates of the reproductive cycle of the aplacental viviparous dogfish. Squalus acanthias Gen Comp Endocrinol.

[27] Koob TJ, Tsang P, Callard IP (1986). Plasma estradiol, testosterone, and progesterone levels during the ovulatory cycle of the skate (*Raja erinacea*). Biol Reprod.

[28] Knight DP, Feng D, Stewart M (1996). Structure and function of the salachian egg case. Biol Rev.

[29] Schindelin J, Arganda-Carreras I, Frise E, Kaynig V, Longair M, Pietzsch T (2012). Fiji: an open-source platform for biological-image analysis. Nat Methods.

[30] Iki A, Anderson WG, Deck CA, Ogihara MH, Ikeba K, Kataoka H, Hyodo S (2020). Measurement of 1α hydroxycorticosterone in the Japanese banded houndshark, *Triakis scyllium*, following exposure to a series of stressors. Gen Comp Endocrinol.

[31] Kobayashi M, Furukawa K, Kim MH, Aida K (1997). Induction of male-type gonadotropin secretion by implantation of 11-ketotestosterone in female goldfish. Gen Comp Endocrinol.

[32] Rasmussen LE, Hess DL, Luer CA (1999). Alterations in serum steroid concentrations in the clearnose skate, *Raja eglanteria*: Correlations with season and reproductive status. J Exp Zool.

[33] Awruch CA, Pankhurst NW, Frusher SD, Stevens JD (2008). Endocrine and morphological correlates of reproduction in the draughtboard shark *Cephaloscyllium laticeps* (Elasmobranchii: Scyliorhinidae). J Exp Zool A.

[34] Knight DP, Feng D (1992). Formation of the dogfish egg capsule: A coextruded, multilayer laminate. Biomimetics.

[35] Feng D, Knight DP (1994). Structure and formation of the egg capsule tendrils in the dogfish *Scyliorhinus canicular*. Phil Trans R Soc Lond B.

[36] Callard IP, Koob TJ (1993). Endocrine regulation of the elasmobranch reproductive tract. J Exp Zool.

[37] Hamlett WC, Knight DP, Pereira FTV, Steele J, Sever DM, Hamlett WC (2005). Oviducal glands in chondrichthyans. Reproductive Biology and Phylogeny of Chondrichthyes: Sharks, Batoids, and Chimaeras.

[38] Threadgold LT (1957). A histochemical study of the shell gland of *Scyliorhinus caniculus*. J Histochem Cytochem.

[39] Rusaouën M (1976). The dogfish shell gland, a histochemical study. J Exp Mar Biol Ecol.

[40] Feng D, Knight DP (1992). Secretion and stabilization of the layers of the egg capsule of the dogfish *Scyliorhinus canicula*. Tissue Cell.

[41] Hamlett WC, Knight DP, Koob TJ, Jezior M, Luong T, Rozycki T (1998). Survey of oviducal gland structure and function in elasmobranchs. J Exp Zool.

[42] Lenain E, Henderson AC (2020). Egg-jelly production and composition in an oviparous chondrichthyan, *Scyliorhinus canicula* (Linnaeus, 1758). J Appl Ichthyol.

[43] Hamlett WC, Musick JA, Hysell CK, Sever DM (2002). Uterine epithelial-sperm interaction, endometrial cycle and sperm storage in the terminal zone of the oviducal gland in the placental smoothhound. Mustelus canis J Exp Zool.

[44] Krishnan G (1959). Histochemical studies on the nature and formation of egg capsules of the shark *Chiloscyllium griseum*. Biol Bull.

[45] Knight DP, Hunt S (1974). Fibril structure of collagen in egg capsule of dogfish. Nature.

[46] Rusaouën M, Pujol JP, Bocquet J, Veillard A, Borel JP (1976). Evidence of collagen in the egg capsule of the dogfish. Scyliorhinus canicula Comp Biochem Physiol.

[47] Mescher AL. Histology and its methods of study. In: Mescher AL, editor. Junqueira’s Basic Histology: Text and Atlas, 12th edition. McGraw Hill, The McGraw-Hill Medical; 2010.p.1–16.

[48] Larson K, Ho HH, Anumolu PL, Chen TM (2011). Hematoxylin and eosin tissue stain in Mohs micrographic surgery: a review. Dermatol Surg.

[49] Knight DP, Feng D, Stewart M, King E (1993). Changes in macromolecular organization in collagen assemblies during secretion in the nidamental gland and formation of the egg capsule wall in the dogfish *Scyliorhinus canicula*. Phil Trans R Soc Lond B.

[50] Feng D, Knight DP (1994). The effect of pH on fibrillogenesis of collagen in the egg capsule of the dogfish. Scyliorhinus canicular Tissue Cell.

[51] Yoshimura Y, Bahr JM, Okamoto T, Tamura T (1993). Effects of progesterone on the ultrastructure of preovulatory follicles of hypophysectomized chickens: possible roles of progesterone in the regulation of follicular function. Jpn Poult Sci.

[52] Ben-Yehoshua LJ, Lewellyn AL, Thomas P, Maller JL (2007). The role of *Xenopus* membrane progesterone receptor β in mediating the effect of progesterone on oocyte maturation. Mol Endocrinol.

[53] Manzano C, Benzal MG, Zelarayan LI (2018). Dynamics of steroid-induced oocyte maturation in three amphibian species: Mathematical modeling and simulation. J Exp Zool A.

[54] Cuellar HS (1979). Disruption of gestation and egg shelling in deluteinized oviparous whiptail lizards *Cnemidophorus uniparens* (Reptilia: Teiidae). Gen Comp Endocrinol.

[55] Conneely OM, Mulac-Jericevic B, Lydon JP (2003). Progesterone-dependent regulation of female reproductive activity by two distinct progesterone receptor isoforms. Steroids.

[56] Gellersen B, Fernandes MS, Brosens JJ (2009). Non-genomic progesterone actions in female reproduction. Hum Reprod Update.

[57] Bahr JM, Wang SC, Huang MY, Calvo FO (1983). Steroid concentrations in isolated theca and granulosa layers of preovulatory follicles during the ovulatory cycle of the domestic hen. Biol Reprod.

[58] Albrecht ED, Pepe GJ, Plant TM, Zeleznik AJ (2014). Placental endocrine function and hormone action. Knobil and Neill’s Physiology of Reproduction.

[59] Cha J, Dey SK, Lim HJ, Plant TM, Zeleznik AJ (2014). Embryo implantation. Knobil and Neill’s Physiology of Reproduction.

[60] Koob TJ, Callard IP (1985). Effect of progesterone on the ovulatory cycle of the little skate *Raja erinacea*. Bull Mt Desert Isl Biol Lab.

[61] Dulvy NK, Pacoureau N, Rigby CL, Pollom RA, Jabado RW, Ebert DA (2021). Overfishing drives over one-third of all sharks and rays toward a global extinction crisis. Curr Biol.

