# Supplementary material for: Progesterone initiates tendril formation in the oviducal gland during egg encapsulation in cloudy catshark (Scyliorhinus torazame)
Source: Zoological Lett. 2023 May 30;9:13. doi: 10.1186/s40851-023-00211-y (PMC10230700; doi:10.1186/s40851-023-00211-y)
Supplement: Supplementary file 1 — Additional file 1: Table S1. Plasma steroid levels of hormone-implanted individuals and morphometry of formed tendrils. [file 40851_2023_211_MOESM1_ESM.pdf]

Supplementary Table 1 Plasma steroid levels of hormone-implanted individuals and morphometry of formed tendrils.

Individual number	P4 (ng/mL)		T (ng/mL)		E2 (ng/mL)		Tendril	
	Before	After	Before	After	Before	After	Length (cm)	Diameter (μm)
No.1 (P4 for 1 day)	0.4	88.2	3.1	6.2	41.5	34.7	2.9	66.2
No.2 (P4 for 1 day)	1.5	69.6	0.4	1.3	1.1	3.1	1.2	19.9
No.3 (P4 for 1 day)	0.4	83.9	0.8	2.3	15.2	11.8	3.0	37.9
No.4 (P4 for 1 day)	0.2	87.1	3.1	3.6	17.0	16.5	4.0	80.0
No.5 (P4 for 2 days)	1.2	73.4	3.7	2.9	28.1	9.8	No data	No data
No.6 (P4 for 2 days)	2.2	98.8	1.4	2.1	7.4	8.9	No data	No data
No.7 (P4 for 2 days)	0.2	163.9	0.5	2.6	26.5	29.2	15.0	267.7
No.8 (P4 for 2 days)	3.7	61.8	9.2	4.5	24.5	17.8	20.0	453.4
No.9 (P4 for 2 days)	0.6	64.7	13.5	13.2	61.9	44.2	23.0	436.7
No.10 (P4 for 5 days)	6.9	67.5	30.7	26.1	47.2	21.1	61.0	156.9
No.11 (P4 for 5 days)	0.5	42.7	1.0	1.2	9.3	3.6	74.5	147.9
No.12 (P4 for 5 days)	1.7	51.1	4.5	4.6	35.0	5.7	67.6	198.0

No.13 (P4 for 2 days using 2 cm tubing)	0.4	8.9	4.6	1.4	10.7	4.6	18.6	183.4
No.14 (P4 for 2 days using 2 cm tubing)	0.1	38.2	13.4	5.1	34.2	19.9	24.8	514.2
No.15 (P4 for 2 days using 2 cm tubing)	0.9	23.1	3.2	4.6	30.8	22.3	15.0	150.1
No.16 (V for 2 days)	1.3	0.6	4.2	7.4	24.0	19.1	Not formed.	
No.17 (V for 2 days)	1.2	2.0	2.4	1.4	15.9	10.1	Not formed.	
No.18 (V for 2 days)	1.0	0.7	15.4	15.1	15.5	17.1	Not formed.	
No.19 (T 2days)	1.1	0.7	0.5	70.1	8.5	16.3	Not formed.	
No.20 (T 2days)	1.1	0.5	10.0	181.6	9.7	14.9	Not formed.	
No.21 (T 2days)	1.0	1.5	22.3	231.7	55.0	55.8	Not formed.	
No.22 (E2 2days)	0.6	0.2	9.0	14.8	27.5	56.0	Not formed.	
No.23 (E2 2days)	1.0	0.1	5.3	6.9	29.6	59.5	Not formed.	
No.24 (E2 2days)	0.7	0.8	14.0	12.4	41.9	58.3	Not formed.	
No.25 (E2 2days)	15.4	11.3	5.2	6.5	25.3	42.6	22.5	778.4
