# Supplementary material for: Progesterone initiates tendril formation in the oviducal gland during egg encapsulation in cloudy catshark (Scyliorhinus torazame)
Source: Zoological Lett. 2023 May 30;9:13. doi: 10.1186/s40851-023-00211-y (PMC10230700; doi:10.1186/s40851-023-00211-y)
Supplement: Supplementary file 2 — Additional file 2: Table S2. Plasma steroid levels of individuals sampled during the endogenous P4 surge and diameters of formed tendrils. [file 40851_2023_211_MOESM2_ESM.pdf]

Supplementary Table 2 Plasma steroid levels of individuals sampled during the endogenous P4 surge and diameters of formed tendrils.

Individual number	Steroid concentrations (ng/mL)				Diameter of tendril (μm)
	The day before sampling	The day of sampling			
	P4	P4	T	E2	
No.26 on the first day of the P4 surge	0.2	33.8	2.2	37.9	45.7
No.27 on the first day of the P4 surge	0.5	24.2	1.7	30.1	36.8
No.28 on the first day of the P4 surge	0.9	24.0	1.5	17.5	53.8
No.29 on the first day of the P4 surge	1.2	26.4	1.8	29.0	36.7
No.30 on the 3 days after the P4 surge		2.2	10.8	26.5	354.0
No.31 on the 3 or 4 days after the P4 surge		6.2	1.9	13.6	331.7
No.32 on the 3 or 4 days after the P4 surge		6.3	8.0	24.4	585.8
No.33 on the 3 days after the P4 surge		8.2	1.5	12.6	681.8
No.34 on the 3 or 4 days after the P4 surge		8.9	2.1	15.5	314.4
