# Supplementary material for: Progesterone initiates tendril formation in the oviducal gland during egg encapsulation in cloudy catshark (Scyliorhinus torazame)
Source: Zoological Lett. 2023 May 30;9:13. doi: 10.1186/s40851-023-00211-y (PMC10230700; doi:10.1186/s40851-023-00211-y)
Supplement: Supplementary file 3 — Additional file 3: Table S3. Occurrence of egg-laying in each individual during implantation. [file 40851_2023_211_MOESM3_ESM.pdf]

Supplementary Table 3 Occurrence of egg-laying in each individual during implantation.

Individual number	Occurrence of egg-laying
No.1 (P4 for 1 day)	No
No.2 (P4 for 1 day)	No
No.3 (P4 for 1 day)	On day 1 of P4 implantation
No.4 (P4 for 1 day)	No
No.5 (P4 for 2 days)	On day 1 of P4 implantation
No.6 (P4 for 2 days)	No
No.7 (P4 for 2 days)	No
No.8 (P4 for 2 days)	No
No.9 (P4 for 2 days)	On day 1 of P4 implantation
No.10 (P4 for 5 days)	No
No.11 (P4 for 5 days)	On day 1 of P4 implantation
No.12 (P4 for 5 days)	On day 2 of P4 implantation
No.13 (P4 for 2 days using 2 cm tubing)	On day 2 of P4 implantation
No.14 (P4 for 2 days using 2 cm tubing)	On day 2 of P4 implantation
No.15 (P4 for 2 days using 2 cm tubing)	No
No.16 (vehicle for 2 days)	No
No.17 (vehicle for 2 days)	No
No.18 (vehicle for 2 days)	No
No.19 (T for 2 days)	No
No.20 (T for 2 days)	No
No.21 (T for 2 days)	No
No.22 (E2 for 2 days)	On day 2 of E2 implantation
No.23 (E2 for 2 days)	No
No.24 (E2 for 2 days)	No
No.25 (E2 for 2 days)	On day 2 of E2 implantation
